# Equine Social Behaviour: Love, War and Tolerance

**DOI:** 10.3390/ani13091473

**Published:** 2023-04-26

**Authors:** Laura Torres Borda, Ulrike Auer, Florien Jenner

**Affiliations:** 1 Equine Surgery Unit, University Equine Hospital, Department of Companion Animals and Horses, University of Veterinary Medicine Vienna, Veterinaerplatz 1, 1210 Vienna, Austria; laura.torres-borda@vetmeduni.ac.at; 2 Anaesthesiology and Perioperative Intensive Care Medicine Unit, Department of Companion Animals and Horses, University of Veterinary Medicine Vienna, Veterinaerplatz 1, 1210 Vienna, Austria; ulrike.auer@vetmeduni.ac.at

**Keywords:** horse, equine, ethogram, social behaviour, sociality, welfare, quality of life

## Abstract

**Simple Summary:**

Horses are highly social animals that preferably live in stable social groups and form long-term affiliative bonds. However, although their need for social interaction has not changed with domestication, domestic horses are often housed in individual stables with limited social contact with other horses or in group housing with regular changes in their group composition. Thus, this review aims to provide an overview of social ethograms to facilitate the inclusion of social behaviour in equine welfare assessment. A literature review yielded 27 papers that studied equine adult social behaviour using a well-defined ethogram. Social interactions were observed in 851 horses living in groups of 9.1 (mean +/− 6.8 s.d., range: 2–33) horses. A total of 40 (mean: 12.8/paper, range: 2–23) social behaviours were described, of which 60% (24/40) were agonistic, 30% (12/40) affiliative, 7.5% (3/40) investigative and 2.5% (1/40) neutral. The 27 papers focused predominantly on socio-negative interactions by including 67.7% agonistic and only 26% affiliative, 5.1% investigative and 1.2% neutral social behaviours in their research. The strong emphasis on agonistic behaviour contrasts sharply with the rarity of agonistic behaviour in stable horse groups and the well-established importance of affiliative interactions for equine welfare. Therefore, to advance the assessment of horses’ welfare, the ethogram needs to be refined to reflect the nuanced and complex equine social behaviour better and consider more affiliative and also ambivalent and socially tolerant interactions.

**Abstract:**

Sociality is an ethological need of horses that remained unchanged by domestication. Accordingly, it is essential to include horses’ social behavioural requirements and the opportunity to establish stable affiliative bonds in equine management systems and welfare assessment. Thus, this systematic review aims to provide an up-to-date analysis of equine intraspecific social ethograms. A literature review yielded 27 papers that met the inclusion criteria by studying adult (≥2 years) equine social behaviour with conspecifics using a well-defined ethogram. Social interactions were observed in 851 horses: 320 (semi-)feral free-ranging, 62 enclosed (semi-)feral and 469 domesticated, living in groups averaging 9.1 (mean +/− 6.8 s.d., range: 2–33) horses. The ethograms detailed in these 27 studies included a total of 40 (mean: 12.8/paper, range: 2–23) social behaviours, of which 60% (24/40) were agonistic, 30% (12/40) affiliative, 7.5% (3/40) investigative and 2.5% (1/40) neutral. The 27 publications included 67.7% agonistic and only 26% affiliative, 5.1% investigative and 1.2% neutral social behaviours in their methodology, thus focusing predominantly on socio-negative interactions. The strong emphasis on agonistic behaviours in equine ethology starkly contrasts with the rare occurrence of agonistic behaviours in stable horse groups and the well-established importance of affiliative interactions for equine welfare. The nuanced and complex equine social behaviour requires refinement of the ethogram with a greater focus on affiliative, ambivalent and indifferent interactions and the role of social tolerance in equine social networks to advance equine welfare assessment.

## 1. Introduction

Horses are gregarious animals that, under naturalistic conditions, spend most of their time in close contact with conspecifics and live in social groups of typically five to six individuals [1,2,3,4,5,6,7,8,9,10,11,12,13,14,15,16,17,18,19]. Harem groups, consisting of one stallion and several mares with their juvenile offspring up to 2–3 years of age, usually have stable adult membership underpinned by long-term social bonds that are established and maintained by affiliative behaviours such as proximity or mutual grooming [3,4,5,6,7,8,9,10,11,12,13,14,15,16,17,18,19,20,21,22,23,24,25,26,27]. Horses show a marked preference for associating with particular individuals, their preferred partners, in their group, with familiarity and homophily counting among the most pervasive factors determining these reciprocal affiliative relationships [14,15,22,25,26,27,28,29,30,31,32,33,34]. Both male and female offspring disperse from their natal group around puberty [7,16,21,32,35]. Despite social dispersal, mares remain spatially philopatric and establish group fidelity to a new harem, typically in proximity to their natal group, at around 3–4 years of age [16]. Dispersed males join bachelor groups that are characterized by a fission-fusion structure [3,21,36,37,38,39]. Solitary horses are only rarely seen, as even displaced older stallions that have lost their harem tend to join bachelor groups [12].

Horses’ social organization is based on a stable, complex dominance hierarchy reflecting resource-holding potential, and a female defence polygyny [4,6,7,11,15,21,22,26,31,32,40]. Equine groups have overlapping home ranges and aggregate, forming multilevel societies (herds) with synchronized daily movement and seasonal migration and stable spatial and hierarchical positioning of the various groups within the herd [17,41]. The social complexity of maintaining long-term affiliative relationships and navigating multilevel societal structures requires the ability to recognize and remember individuals and their relative rank [17,42,43]. Indeed, horses are capable of cross-modal individual recognition using visual, auditory and olfactory cues even after a year’s absence and transitive inference of dominance relationships through observation [44,45,46,47,48,49,50,51]. Horses’ social cognition is further demonstrated by third-party interventions in agonistic and affiliative dyadic interactions of group members and increased affiliative behaviour after a conflict [34,52,53]. As food-related aggression is not typically relevant in grazers that feed on widely dispersed and undefendable resources, agonistic interactions occur mainly to establish a dominance hierarchy and maintain personal space, in which horses only allow affiliative associates [13,14,39,54]. Dominance typically depends on age, physical characteristics, experience, and length of residency in the herd [13,14,39,55]. The stable composition and hierarchy of (semi-)feral equine groups and the long-term social bonds result in social cohesion and a low frequency of agonistic interactions, most (80%) of which are ritualized and do not involve physical contact [54,56].

Comparisons of the behaviour of feral and domesticated horses indicate that the species-specific social behaviour of horses has remained qualitatively relatively unchanged by domestication [13]; however, the environment of domestic horses has changed dramatically compared to naturalistic conditions. Although management systems that accommodate equine sociality exist, most domestic horses are confined to individual stables with limited contact with conspecifics [12,13,15,22,35,56,57,58,59,60,61,62,63,64,65,66]. Lack of social contact is thought to be one of the most serious stressors for horses, as evidenced by significant increases in faecal corticosterone metabolites, and it triggers stress-related behaviours and stereotypies such as weaving, cribbing and box-walking in horses kept without adequate opportunities to socialize with conspecifics [12,54,56,58,60,67,68,69,70,71,72,73,74,75,76,77,78]. Indeed, social contact, specifically the possibility to engage in affiliative behaviours such as allogrooming, which has been shown to lower the heart rate, has been identified as an ethological need and essential for equine welfare [15,22,56,79]. In addition to the limitation in social contact, managed horses also do not have the opportunity to choose their group affiliation. They are faced with frequent changes in group composition and social companionship, which limits their opportunities to establish long-term social bonds and a stable hierarchy, resulting in higher aggression and frequency of agonistic encounters [56,58,63,80]. The space restrictions inherent to domestic conditions, which limit the opportunities for subordinate individuals to escape or provide dominant conspecifics with their required individual distance, further compound the social challenge [56,58,62,80,81]. As horses do not adapt to repeated regrouping and a stable hierarchy is achieved only after 2–3 months [56,58], the common disregard of equine social group dynamics in equine husbandry poses a significant welfare concern [12,56,57,58,61,62,81]. 

Thus, it is essential to include horses’ social behavioural needs and the opportunity to establish stable affiliative bonds in equine management systems and welfare assessment [82]. However, to facilitate evidence-based optimization of equine husbandry practices and their evaluation, the influence of different environmental and management factors on equine social interactions needs to be further elucidated [83]. As differences in the sampled behaviours currently complicate the comparison of equine social behavioural studies, this systematic review aims to analyse the literature on equine social ethograms to distil a well-defined social behavioural repertoire as a basis for further studies. 

## 2. Materials and Methods

### 2.1. Data Sources and Searches

**Figure 1:**
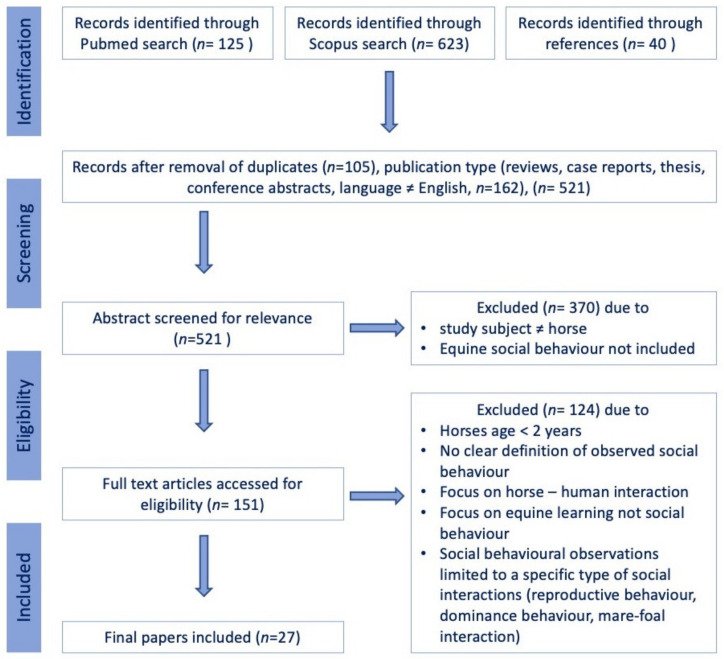
Flow chart illustrating the study selection process for the systematic review.

This review was carried out according to the Preferred Reporting Items for Systematic Reviews and Meta-Analyses (PRISMA) guidelines [84]. Scientific peer-reviewed articles focused on adult (≥2 years) equine intraspecific social behaviour were identified through a systematic search in the PubMed (National Institutes of Health. PubMed [Database]. Bethesda, MD, USA: National Library of Medicine; https://pubmed.ncbi.nlm.nih.gov, accessed on 25 January 2022) and Scopus (Elsevier, Amsterdam, The Netherlands; https://www.scopus.com) electronic databases on 25 January 2022. The search was conducted by combining the search strings (“horse” OR “equine” OR “equus”) in the title and (“social” OR “ethogram” OR “agonistic” OR “affiliative”) in the title or abstract with the Boolean operator “AND”, with no restriction on publication date. The following exclusion criteria were set a priori (Figure 1): (a) non-peer-reviewed publication, dissertation, thesis, review, commentary, or single case report; (b) only a conference/seminar abstract published; (c) the article was not written in English; (d) the study did not include equine intraspecific social behaviour but focused on interspecies interaction or learning behaviour; (d) no ethogram of observed social behaviour was provided; (e) the observations were limited to a specific subset of behaviours (reproductive behaviour, dominance behaviour, mare-foal interaction); or (f) the observed horses were <2 years of age.

### 2.2. Data Extraction and Risk of Bias Assessment

The study selection process was carried out by U.A. and F.J. following the procedure detailed in Figure 1. Any disagreement between the authors on the studies included in the review was resolved during a consensus meeting.

Information on the population, intervention, comparison, outcome and study design (PICOS) was retrieved from the articles, and the risk of bias in selected studies was assessed using a modification of the Evidence Project risk-of-bias tool [85,86,87].

## 3. Results

### 3.1. Study Selection

A total of 125 articles were identified in PubMed, 623 additional papers in Scopus and another 40 based on references, yielding a total of 788 articles (Figure 1). After removing duplicates, reviews, commentaries, single case reports, books and non-English or German articles, 521 papers remained. Following the exclusion of papers that did not focus on adult (≥2 years) equine intraspecific social behaviour but on interspecies interaction or other behavioural observations or did not provide a well-defined ethogram of the observed behaviours, 27 articles remained and were included in the qualitative synthesis [5,12,13,14,22,26,27,29,30,34,37,41,52,54,55,56,58,62,79,80,88,89,90,91,92,93,94].

### 3.2. Quality and Risk of Bias Assessment

Of the 27 included papers, 22 (81.5% of the total) were ecological observational studies [5,12,14,22,26,27,30,34,37,41,52,54,55,56,62,79,88,89,90,91,92,93], and 5 (18.5%) prospective, non-blinded experimental studies, of which 3 had a pre-post [29,80,94] and 2 a randomized trial design [13,58]. Measurements of the dependent variables were conducted before and after a specific intervention, such as a change in paddock size [94], feeding tests [29] and a controlled change in a group composition [58]. 

Risk-of-bias assessment (Table 1) revealed the lack of a control group (only 7.4% of the articles fulfilled this criterion) [13,58], random assignment of participants to intervention (7.4% of the articles fulfilled this criterion) [13,58], a random selection of participants for assessment (none of the articles fulfilled this criterion), as the most critical concerns. Further limitations of some papers were caused by lacking control over confounding variables, such as changes in groups’ composition that were not controlled by the researchers [62,93] and specific interventions that were not part of the study design but may impact horses’ behaviour (e.g., mating of individuals during the study [29]; riding [94]).

The number of horses (6–145 horses/paper, mean: 31.5, +/− 32.5 s.d.), groups (1 to 18 groups, mean: 3.1, +/− 4 s.d.) and group size (2 to 33 individuals, mean: 9.1, +/− 6.8 s.d.) varied considerably between papers. The observation methods were restricted to direct manual observation in the field (27/27 papers, 100%), with additional manual behaviour scoring from video carried out in only 11.1% of the studies (3/27) [56,80,94]. No study used biotelemetry devices. The expression of specific social behaviours was assessed using four different methods: focal sampling in 18 (66.67%) papers [5,12,13,14,26,29,30,52,54,58,79,80,88,89,91,92,93,94]; ad libitum sampling in 6 (22.22%) papers [27,30,34,37,41,52]; all occurrence sampling in 4 (14.81%) papers [29,55,62,90]; and behaviour sampling in 1 (3.70%) [56]. In addition, scan sampling was applied in 59.26% (16/27 studies) to investigate spatial patterning [5,12,13,14,22,26,27,29,30,34,41,54,62,79,89,92]. 

Most studies (92.59%, 25/27) conducted their observations exclusively during the day (6:00–19:30) [5,13,14,26,27,29,30,34,37,41,52,54,56,58,62,79,80,88,89,90,91,92,93,94]; two (7.41%) included evening hours (up to 12 a.m.) [12,55], and only one observed the horses for the entire day (0–24 h) [22]. The observation time per group ranged from less than 6 h during an observation day in 59.26% (16/27) [12,13,14,29,30,52,54,55,58,79,80,88,89,90,91,94]; 6 to 12 h in 18.52% (5/27) [5,34,41,56,92]; and 24 h in 3.7% (1/27) [22]. The exact observation times were not provided in 14.81% (4/27) of the articles [27,30,37,93] and varied between 3 and 8 h a day for one article [62]. 

### 3.3. Data Synthesis

Six papers (22.2% of the total) studied free-ranging (≥300 ha) (semi-)feral horses [5,27,34,41,55,94]; six (22.2% of the total) (semi-)feral horses living in enclosures ranging from 2800 m^2^ to 75 ha [13,52,79,90,91,92]; and seventeen (58.6% of the total) domesticated horses [12,13,14,22,26,29,30,37,54,56,58,62,80,88,89,93,94] housed in paddocks or pastures ranging in size from 160 m^2^ to 17.2 ha. Two papers compared the behaviour of domesticated and semi-feral horses [13,93]. 

A total of 851 horses, aged 2–32 years, are included in the present systematic review (Table 2), of which 320 were free-ranging (semi-)feral horses, 62 enclosed (semi-)feral horses and 469 domesticated horses. The studies included, on average, 31.5 horses (+/− 32.5 s.d., range: 6–145) overall; 53.3 (+/− 53.9 s.d., range: 8–145) free-ranging (semi-feral) horses; 10.3 (+/− 3.8 s.d., range: 6–16) enclosed (semi-)feral horses; and 27.6 (+/− 22.7 s.d., range: 9–78) domesticated horses. 

The average number of groups per paper was 3.1 (+/− 4 s.d., range: 1–18) overall; 3.8 (+/− 3.65 s.d., range: 1–11) for free-ranging (semi-)feral horses; 1.2 (+/− 0.4 s.d., range: 1–2) for enclosed (semi-) feral horses; and 3.2 (+/− 4.5 s.d., range: 1–18) for domesticated horses. Group size averaged 9.1 horses/group (+/− 6.8 s.d., range: 2– 33) overall; 13.9 (+/− 8 s.d., range: 4–30) for free-ranging (semi-feral) horses; 8.9 (+/− 4.5 s.d., range: 4–16) for enclosed (semi-)feral horses; and 8.6 (+/− 7.2 s.d., range: 2–33) for domesticated horses.

The ethograms detailed 40 non-redundant intraspecific social behaviours (mean: 12.81/paper +/− 4.6 s.d., range. 2–23) (Table 3). Seven papers (25.93%) included less than ten different behaviours [5,27,29,80,88,91,93], thirteen papers (48.15%) between 10 and 15 different behaviours [12,13,14,22,26,30,34,41,52,56,62,89,94] and seven papers (25.93%) more than 15 behaviours [37,54,55,58,79,90,92]. The 40 social behaviours encompassed 24 agonistic interactions (60%), of which 19 were aggressive (47.5%) and 5 submissive (12.5%), but only 12 affiliative (30%), 3 investigative (7.5%) and one “neutral” behaviour (2.5%) (Table 3). Analysis of the application of the social ethogram revealed that the 27 papers detailed specific social behaviours as part of their methodology 331 times, of which 224 (67.7%) were agonistic, 86 (26%) affiliative, 17 (5.1%) investigative and 4 (1.2%) neutral behaviours, further confirming the focus on agonistic behaviours in equine ethology.

The definitions of the social behaviours were similar between the different studies with only subtle differences between papers (e.g., “retreat” [27,34,37,41,52,80] and “avoidance” [14,22,26,30,37,62,90,92] were used interchangeably, “displacement” [54,62] was used to describe “supplantation” [29,79] and “agonistic approach eliciting retreat” [12,13,14,26,27,29,30,34,37,41,52,55,58,91,92]). Some papers limited the definitions of the ethograms to few words, making them more concise but also less precise and hence ambiguous [14,30,90], which can lead to confusion in the distinction of similar behaviours (e.g., “mild-threat” [14,30] and “head-threat” [5,29,37,62,79,90,94]) or behavioural patterns (e.g., “agonistic approach” [12,13,14,26,27,29,30,34,37,41,52,55,58,91,92] versus “displacement” [54,62] versus “supplantation” [29,79]).

In addition to the qualitative description of social behaviour, 22 papers (81.5%) also quantified social interactions (Table 4), including the frequency of behaviours and proximity events, the duration of interactions, ranking and dominance relationships [5,12,13,14,22,27,29,34,37,52,54,55,56,58,62,79,80,89,90,91,92,93,94]. Two articles included network analyses [41,52].

## 4. Discussion

Aiming to provide an up-to-date analysis of equine social ethograms, this systematic review included all original studies of equine social behaviour that detailed the ethogram underlying the reported research. Surprisingly, the equine social ethograms primarily reference the “agonistic ethogram of the equine bachelor band” (includes 23/40 behaviours, is referenced in 15/27 papers [12,13,22,27,34,37,41,52,54,56,58,80,92,93,94]), which was based on a literature review of equine behaviours and 50 daylight hours of observation of 15 pony stallions (2–21 years old) pastured together in a semi-natural enclosure of 9 acres [37]. While this landmark publication provides an excellent ethogram, it describes interactions in an equine bachelor group, which is neither under (semi-)feral nor domestic conditions the prevalent social group structure and hence may not suffice as a comprehensive behavioural catalogue for horses living in harems or human-managed groups. The strong focus on agonistic behaviours has persisted in equine ethology, with 67.7% of the social behaviours studied in the 27 papers focussing on the socio-negative spectrum and only 26% on affiliative, 5.1% on investigative and 1.2% on neutral behaviours. The rare occurrence of agonistic behaviours in stable horse groups (0.2–1.5 agonistic interactions/h per horse [13,56,79,95,96,97] and the well-established importance of affiliative interactions for equine welfare [12,15,22,31,54,56,58,60,67,68,69,70,71,72,73,74,75,76,77,78,79] further emphasize the necessity to expand and diversify the equine social ethogram to include a broader spectrum of behaviours of horses living in different group compositions and environments. 

The binary division in agonistic and affiliative social interactions as bipolar opposites belies the reality that many relationships are not one-dimensionally positive or negative but more multifaceted and may entail social tolerance, coactivated feelings of positivity and negativity toward a relational partner (ambivalence) or lack affective valence (indifference) [80,90,98,99,100,101,102,103,104,105,106,107,108]. In human social sciences, the impact of ambivalent relationships on social networks and quality of life is increasingly recognized. Studies have shown them to be prevalent in both personal and professional networks and cause increased stress, blood pressure and detrimental health outcomes [98,109,110]. The social behaviour of horses similarly includes ambivalent interactions and relationships, such as the more frequent but less violent aggressive interactions among preferred associates and the predominant initiation of affiliative interactions by dominant individuals [5,22,26,29,30,56,80,90,107]. In addition, horses show social tolerance (defined as proximity to conspecifics around valuable resources with little or no aggression [108]) depending on space availability and their social experience [5,22,26,29,30,56,80,90,107]. As the current positive–negative dichotomy does not sufficiently reflect the nuanced and complex equine social behaviour, equine ethology can build upon the human social science approach of assessing the valence generated by social interactions along the continuum from negative to positive in combination with the elicited autonomic activation intensity (arousal) to refine equine ethograms [98,99,110,111,112,113,114]. 

The quantification of social interactions incorporated by 81.55% of the included papers also assists in assessing dyadic relationships and group dynamics. These quantitative criteria are primarily based on the reported interindividual distance of 2 m to two horse lengths, within which horses only tolerate close affiliates [12,13,27,38,39,54,115,116,117] and include measurements of spatial proximity between two horses, the number and duration of affiliative or agonistic interactions per hour and recording the nearest neighbour. In addition, recent studies have incorporated social network analysis to examine indirect connections beyond the dyad level and analyse the patterns of individual and group-level social interactions [41,52,118,119,120,121]. 

The combination of a more nuanced qualitative assessment of equine social behaviour with quantitative approaches may greatly assist equine welfare assessment and optimization, as poor welfare conditions, such as high population density (< 331 m2 per horse), may reduce equine sociality and skew horses’ social behavioural repertoire toward agonistic interactions [80,122,123]. Changes in social behaviour have also been associated with disease in various group-living species ranging from honeybees, zebrafish and mice to calves and humans [121,124,125,126,127], but the link between social networks and health has not yet been explored for horses. The changes seem species-specific, as mice reduce social interactions, while rhesus macaques and calves increase affiliative interactions with familiar conspecifics [121,128,129]. More detailed studies in sick humans found increased social interactions with familiar support figures but withdrawal from strangers and a strong correlation between pain intensity and interpersonal distance to strangers in patients with lower back pain [121,127,130,131]. Expanding social behavioural research in horses to also include assessment of the effect of acute and chronic disease on social interactions may further advance equine welfare by facilitating early detection, treatment and monitoring of disease and pain. 

## 5. Conclusions

Horses, as social non-territorial equids that preferably live in stable, hierarchically structured social groups, have developed complex cognitive skills, ritualized communication signals and nuanced social behaviour. In these stable groups, the frequency of agonistic interactions is low under species-appropriate housing and welfare conditions (e.g., adequate enclosure size, stocking density and resource availability). However, our systematic review reveals a strong focus of current social ethograms on socio-negative interactions with 67.7% agonistic and only 26% affiliative, 5.1% investigative and 1.2% neutral social behaviours. The traditional equine ethology approach focusing on univalently negative social interactions does not sufficiently reflect the complexity of equine social behaviour and requires the development of a more refined ethogram, which also considers ambivalent and indifferent interactions and relationships and the role of social tolerance in equine social networks. A standardized comprehensive social ethogram combined with quantification of social interactions and social network analysis would facilitate research into the effect of disease and pain on equine social behaviour and constitute a valuable tool for equine welfare assessment and optimization.

## Figures and Tables

**Table 1:** List of the included articles, their study design, observation method(s) and the number of observation days.

Author(s), Publication Year	Study Design	Control Group	Observation Method(s)	Observation Duration	Observation Time Window	Number of Incl. Behaviours
Wells & Goldschmidt-Rothschild 1979 [5]	Observational study (field)-ecological study design	Absent. No randomization for group constitution	Random order, direct in the field, focal and scan sampling (15 min 3×/day per horse)	16 weeks (4 × 3 blocks, 1 × 4-week block)	7:00–19:00	8
Arnold & Grassia 1982 [88]	Observational study (field)-ecological study design	Absent. No randomization for group constitution	Random order, direct in the field, focal sampling (4 h/day)	Between October and December	2 h in the morning and 2 h in the afternoon	2
Wood-Gush & Galbraith 1987 [89]	Observational study (field)-ecological study design	Absent. No randomization for group constitution	Random order, direct in the field, focal and scan sampling (1×/h or 1×/15 min all positions + 15 min continuously of activity and social interaction)	11.5 weeks (36 h)	8:30–16:30	10
Feh 1988 [79]	Observational study (field)-ecological study design	Absent. No randomization for group constitution	Direct in the field, focal and scan sampling (1×/10 min/horse all positions)	4 h/day, 5 weeks	8:00–19:00	17
Keiper 1988 [91]	Observational study (field)-ecological study design	Absent. No randomization for group constitution	Random order, direct in the field, focal sampling (15 min/horse)	2 months, 44.5 h in total	4–5 h, between 9:00 and 16:00	9
Kolter & Zimmermann 1988 [90]	Observational study (field)-ecological study design	Absent. No randomization for group constitution	Random order, direct in the field, all occurrence sampling technique	113 h in total, throughout the year	2 h in the morning + 2 h in the afternoon	16
Ellard & Crowell-Davis 1989 [29]	Experimental study—Pre-post study design. Randomized pairs for testing	Absent. No randomization for group constitution	Direct in the field, all occurrence sampling techniques and scan sampling (nearest neighbour every 15 min)	56.7 h in total (2 h/day, 5 d/week, 6 weeks)	15.00–17.00	9
Keiper & Receveur 1992 [55]	Observational study (field)-ecological study design	Absent. No randomization for group constitution	Direct in the field, all occurrence sampling techniques	159 h in total (4.5 h/day, 41 days)	4 or 5 h, between 5:00 and 24:00	16
McDonnell & Haviland 1995 [37]	Observational study (field)-ecological study design	Absent. No randomization for group constitution	Direct in the field, ad libitum sampling technique	50 h in total, 4 weeks	daylight hours	23
Christensen et al., 2002 (a) [12]	Experimental study—Randomized controlled trial study design	Horses randomly assigned, individual vs. group stabling	Direct in the field, focal sampling (social interaction: 3 h/day/group) and scan sampling (nearest neighbours: every 10 min for 1 h, 4 days/week)	192 h in total, 28 h/week for 6 weeks	3 h, 6:00 and 22:00	14
Christensen et al., 2002 (b) [13]	Observational study (field)-ecological study design	Comparison between two non-randomized groups, no interventions	Direct in the field, focal sampling (social interaction: 3–4 h/day/group) and scan sampling (nearest neighbour: every 10 min)	72 h/group	3 or 4 h windows during daylight hours	14
Snorrason et al., 2003 [22]	Observational study (field)-ecological study design	Absent. No randomization for group constitution	Random order, focal sampling (social interaction: 15 min) and scan sampling (nearest neighbour: every 30 min)	488 h in total, 5 weeks	throughout 24 h	11
Heitor et al., 2006a (part I) [14]	Observational study (field)-ecological study design	Absent. No randomization for group constitution.	Random order, direct in field, focal sampling for social interactions, scan sampling every 5 min for activity and nearest neighbour	386 h, 80.4 h per mare (range 74.9–88.1) and 54.5 h for the stallion	between 07:30 and 16:30 h	14
Heitor et al., 2006b (part II) [30]						14
Jørgensen et al., 2009 [54]	Observational study (farm)-ecological study design	Comparison between three groups with non-randomized composition, no interventions	Direct in the field, focal sampling for social interactions (2 h/day for 3 days, 4 horses/group), scan sampling for nearest neighbour every 10 min	6 h/group	between 8:00–11:00 and 12:00–15:00	18
Zharkikh & Andersen 2009 [92]	Observational study (field)-ecological study design	Absent. No randomization for group constitution	Random order, direct in the field, focal sampling (15 min, 3×/horse/day) and scan sampling for nearest neighbour every 10 min	216 h, 18 days	between 6:00 and 18:00	22
Heitor & Vicente 2010 [26]	Observational study (farm)-ecological study design	Absent. No randomization for group constitution	Random order, direct in the field, focal sampling (social interaction: 25 min/horse/day) scan sampling for nearest neighbour every 5 min and ad libitum	141 h in total, 5 months	between 6:30 and 18:30	15
Christensen et al., 2011 [58]	Experimental study-Randomized controlled trial study design	Horses were randomly assigned, stable group vs. unstable group	Direct in the field, focal sampling (2 × 20 min/group/day)	3 months per year for 2 years	between 8.00–11:00 and 12:00–15:00	16
Schneider & Krueger 2012 [34]	Observational study (field)-ecological study design	Absent. No randomization for group constitution	Direct in the field, ad libidum sampling of third-party interventions and scan sampling (1×/h group spatial map)	44 h over three months (non-consecutive)	daylight hours (max. 6.5 h/day)	11
Flauger & Krueger 2013 [80]	Experimental study—Pre-post study design	Absent. No randomization. Groups measured before and after intervention (change of paddock size)	Focal sampling (4 h/group) and focal sampling (introduction of new horses (2 h/introduction)	variable number of observations between groups (average 6 times, range 1 to 13)	NA	7
Freymond et al., 2013 [56]	Observational study (field)-ecological study design	Absent. No randomization for group constitution	Behaviour sampling of social interactions	23 days: 109 h/horse, 17 days: 87 h/horse	either 9–11; 13–15; 17–18 or 7–9; 11–13; 15–17	14
Krueger et al., 2014 [41]	Observational study (field)-ecological study design	Absent. No randomization for group constitution	Direct in the field, ad libidum sampling (social interaction: 14 h/group) and scan sampling (spatial organization: map drawn 1×/h for 15 h/group	May 2009 and May 2010	daylight hours (max 6.5 h/day)	12
Krueger et al., 2015 [52]	Observational study (field)-ecological study design	Absent. No randomization for group constitution	Direct in the field, ad libidum sampling (social interaction: 9 × 4 h) and focal sampling (newly introduced horse 4 × 2 h/horse)	between April 2008 and May 2010	for 4 h approximatively, daylight hours	11
Górecka-Bruzda et al., 2016 [93]	Observational study (field)-ecological study design	Comparison between two groups with non-randomized composition with no interventions	Przewalski: twice/day during 5 time slots, 10 min/focal horse, 10 h/horse in total Domestic horses: 3 time slots, 5 min/focal horse, 4.16 h/horse in total	Przewalski horses: 10 h/horse; Domestic horses: 4.16 h/horse	Przewalski: daylight hours (7:00–21:00); Domestic Horses: daylight hours (6:00–19:30)	9
Majecka & Klawe 2017 [94]	Experimental study—Pre-post study design	Absent. No randomization. Measurements before and after intervention (=change of paddock size)	Direct in the field, focal sampling (social interaction, 30 min once or twice/group/day)	between March and July 2011, 43 × 30 min	9 a.m.–12 p.m.	13
Wolter et al., 2018 [27]	Observational study (field)-ecological study design	Absent. No randomization for group constitution	Direct in the field, continuous ad libitum sampling (social interaction) and scan sampling (spatial proximity every 10 min)	165 h in total	daylight hours	9
Pierard et al., 2019 [62]	Observational study (field)-ecological study design	Absent. No randomization for group constitution	Direct in the field, all occurrence sampling (social interaction: 90–120 min, 2–4×/day) and scan sampling (spatial position every 15 min)	17 days; 54 h 25 min	not fixed	12

**Table 2:** Signalment of the horses included in the study. Depending on the data available in the respective papers, ages are provided as range, median (plus range), or mean ± standard deviation. Similarly, the sex is detailed depending on the information provided in the papers.

Author(s), Publication Year	Total Horses (*n*)	Number of Herds	Horses/Herd	Type and Breed	Sex	Age (Years) Mean +/− s.d. (Range)	Size of Enclosure	Feeding
Wells & Goldschmidt-Rothschild 1979 [5]	18	1	18	semi-feral (Camargue)	8 mares, 2 stallions, 22-year-old stallions, 6 yearlings, 7 foals	NA	300 ha	NA
Arnold & Grassia 1982 [88]	study 1: 17; study 2: 12	1	study 1: 17; study 2: 12	domesticated (NA)	study1: 16 mares/1 male; study 2: 11 mares/1 male	study 1: 10.3 +/− 7 (3–24); study2: 19.6 +/− 7.5 (4–32)	12 ha, 15 ha	chaff, grains, hay
Wood-Gush & Galbraith 1987 [89]	13	1	13	domesticated (Exmoor, Highland)	2 mares, 11 males	14 +/− 5.2 (6–22; one NA)	2 ha	daily hay
Feh 1988 [79]	9	2	4; 5	semi-feral (Przewalski)	4 mares, 5 males	3 +/− 1.2 (2–5)	4 ha, 16 ha	grass, hay, pellets
Keiper 1988 [91]	6	1	6	semi-feral (Przewalski)	5 mares, 1 male, 3 foals	8 +/− 8.9 (0–21)	NA	NA
Kolter & Zimmermann 1988 [90]	7	1	7	semi-feral (Przewalski)	6 mares, 1 male	9.75 +/− 7.5 (1–22)	2800 m^2^	hay, oats, pellets
Ellard & Crowell-Davis 1989 [29]	12	1	12	domesticated (Belgian, Percheron)	12 mares	6.9 +/− 3.6 (2–13)	10 ha	daily hay
Keiper & Receveur 1992 [55]	10	2	5; 5	semi-feral (Przewalski)	6 mares, 4 stallions	3 +/− 2.8 (0–8)	37 ha, 350 ha	grass, hay, pellets
McDonnell & Haviland 1995 [37]	15	1	15	domesticated (NA)	15 stallions	(2–21)	2 acres	NA
Christensen et al., 2002 (a) [12]	19	2	12; 7	domesticated (Danish Warmblood)	19 stallions	2 years old	group 1: 5.6 × 4.8 m boxes + 40 × 90 m paddocks; group 2: 3.6 × 2.5 m boxes + 20 × 40 m paddocks; 2 ha/group; 4 ha	barley straw, concentrate, grass, hay, molasses, silage
Christensen et al., 2002 (b) [13]	32	1; 1	19; 13	domesticated (NA); semi-feral (Przewalski)	32 stallions	group 1: 2; group 2: 5.2 +/− 3.3 (2–13)	4 ha, 75 ha	grass
Snorrason et al., 2003 [22]	33	1	33	domesticated (Icelandic)	17 mares, 2 geldings, 14 yearlings and 8 foals (sex not specified)	9 +/− 6.7 (1–20)	8 ha	grass, silage
Heitor et al., 2006a (part I) [14]	11	1	11	domesticated (Sorraia)	10 mares, 1 stallion	11 +/− 3.6 (5–18)	5.5 ha, 17.2 ha	grass, hay
Heitor et al., 2006b (part II) [30]	11	1	11	domesticated (Sorraia)	10 mares, 1 stallion	11 +/− 3.6 (5–18)	5.5 ha, 17.2 ha	grass, hay
Jørgensen et al., 2009 [54]	66	3 × 6 rounds	3; 3; 4; 4; 5; 3; 3; 3; 4; 4; 6; 4; 4; 4; 4; 5; 9; 3	domesticated (Warmblood, Norwegian Fjord)	22 mares, 24 males. Composition of one group unspecified	(1–26)	from 100 to 75,000 m^2^/horse	grass, roughage
Zharkikh & Andersen 2009 [92]	16	1	16	semi-feral (Przewalski)	16 males	(5–16)	3.5 ha	grass
Heitor & Vicente 2010 [26]	11	1	11	domesticated (Sorraia)	11 mares	(4–22)	5.5 ha, 17.2 ha	grass, hay
Christensen et al., 2011 [58]	45	15	3	domesticated (Danish Warmblood)	45 mares	2-years-old	80 × 80 m	barley, barley straw, grass, seed cake and minerals, silage
Schneider & Krueger 2012 [34]	84	4	14; 20; 30; 20	feral (Espéra ponies)	group 1: 13 mares/1 stallion; group 2: 19 mares/1 stallion; group 3: 27 mares/3 stallions; group 4: 19 mares/1 stallion	(1–28)	free-ranging	mountain pastures
Flauger & Krueger 2013 [80]	68	11	3; 4; 3; 8; 14; 3; 3; 15; 4; 8; 3	domesticated (Warmblood, Quarter horses, Trotters, Haflingers, ponies)	NA	(1–30)	402 m^2^, 17,882 m^2^	NA
Freymond et al., 2013 [56]	9	2	5; 9 (four were included in both groups)	domesticated (Franches-Montagnes)	9 stallions	(8–19)	4 ha	hay
Krueger et al., 2014 [41]	55	3	11; 19; 25	feral (Espéra ponies)	group 1: 10 mares/1 stallion; group 2: 18 mares/1 stallion; group 3: 22 mares/3 stallions	(1–23)	free-ranging	hay
Krueger et al., 2015 [52]	11	1	11	semi-feral (Przewalski)	11 stallions	(2–8)	50 ha	hay, horse feed
Górecka-Bruzda et al., 2016 [93]	27	2; 4	4–6, 4–6; 5, 2, 8, 4	semi-feral; domesticated	semi-feral groups: 4–6 adult males; domestic group 1: 2 males + 3 geldings; group 2: 2 males; group 3: 1 male + 7 females; group 4: 1 male + 3 females	NA	from 2 to 1600 ha	hay
Majecka & Klawe 2017 [94]	78	3	26; 28; 24	domesticated (Friesian, Arabian, Shetland, Warmblood)	41 mares, 25 geldings, 12 stallions	group 1: 10.2 (2–21); group 2: 8.8 (2 months-30 years); group 3: 5.3 (3 months-16 years)	from 0.35 to 8.1 ha	hay
Wolter et al., 2018 [27]	145	11	5; 7; 6; 9; 9; 23; 10; 12; 19; 26; 19	semi-feral (Przewalski)—feral (Equus ferus caballus)	113 mares, 32 males	group 1: 2.6; group 2: 8.7; group 3: 8.5; group 4: 6.2; group 5: 10.4; NA for other groups	free-ranging	hay
Pierard et al., 2019 [62]	11	1	11	domesticated (Irish Cob, Arabian, Warmblood)	10 mares, 1 gelding	10 +/− 7.3 (1–29)	from 160 m^2^ to 610 m^2^	hay

**Table 3:** Ethograms of adult equine social behaviour used in the 27 papers.

Social Behaviour Category	Social Behaviour	Definition	Differences in the Definition	Used by n/27 Papers	Comments
	Approach eliciting retreat [12,13,14,26,27,29,34,37,41,52,55,58,91,92]/Displacement [54]	Approach of one horse with ears back causes another to move away so that distance is maintained or increased	“Approach within 2 m distance” [29]/two body-length distance14	15	Displacement is used variably either to describe “eliciting retreat” or “supplant”; to minimize ambiguity, we propose avoiding “displace” and differentiate the two types of agonistic approach as approach-retreat and approach-supplant
**agonistic–aggressive**	(Approach with) Supplantation [29,79]/Displacement [64]	Horse moving toward another horse and taking the exact same place after the other horse moved away	Either individual may have laid back ears [29]	3	
	Arched neck threat [37]	Neck tightly flexed with the muzzle drawn toward the chest; observed during close aggressive encounters and ritualized interactions		1	
	Attack [14,22,26,30,90]/Lunge [37,56]	Fast movement toward another horse, with ears flattened, head stretched horizontal	“One horse rears with the forelegs in the direction of another horse, ears laid back” [56]	7	
	Backing [54]	Backward movement towards another horse with ears oriented backwards		1	
	Bite [5,12,13,14,22,26,27,29,30,34,37,41,52,54,55,56,58,64,79,80,88,89,90,91,92,93,94]	Ears are laid back and teeth are closed on some body part of another animal. Lips retracted and contact is made with the target horse		27	Bite is considered as a grasp if the hold is maintained
	Bite threat [5,12,13,14,22,26,27,29,30,34,37,41,52,54,55,56,58,64,80,88,89,90,91,92,93,94]	Ears are laid back, the mouth is opened, and a biting motion is made while head or full body motion toward another animal, no contact is made. A bite intention movement and neck extended		26	
	Chase [14,22,26,27,29,30,34,37,41,52,54,55,56,58,64,80,90,91,92,93,94]	With its ears pinned back, the aggressor chases another individual	Specification of “for at least 1” [29] or “3 [64] strides”; “at a gallop“ [92]; “The movement can be either at a walk, trot or gallop” [80]	21	We propose limiting “chase” to fast gaits to differentiate between “agonistic approach” and “chase”
	Circling [37,56]	Two horses circle each other head-to-tail, trying to nip or bite each other’s body parts		2	Can also be part of play behaviour
	Fight [92]	High-level prolonged mutual aggression involves bites, strikes, kicks, chase, etc. Usually, the opponents squeal		1	
	Head bump [37]	A rapid lateral toss of the head that forcefully contacts the head and neck of another horse. Usually, the eyes remain closed and the ears forward		1	
	Head-threat [5,29,37,54,64,79,90,94]	The extension of the aggressor’s head and neck towards another individual while laying the ears against its head		8	
	Herding [14,26,30,37,41,55,90,91]/Driving [5]/Snaking [92]	Combination of head threat with the ears back and forward locomotion directing the movement of another horse	Swinging head sideways [14,91,92]	10	
	Kick [12,13,14,22,26,27,29,30,34,37,41,52,54,55,56,58,64,79,80,88,89,90,91,92,93,94]	With its ears laid back, one or both hindlegs of the aggressor are extended backwards rapidly and strike another animal with apparent intent to make contact		26	
	Kick-threat [5,12,13,14,22,26,27,29,30,34,37,41,52,54,55,56,58,64,79,80,88,89,90,91,92,93,94]	The aggressor, with its ears laid back, either (1) makes a rapid movement to place its hindquarters near another animal; or (2) raises a hind limb to potentially strike another; or (3) kicks with 1 or both hindlimbs towards another animal, but no contact is made	Vigorous tail switching, production of a harsh squeal [37,93]	27	
	Mild threat [14,26,30]	Ears laid back and looking or walking towards another horse		3	Definition lacks details; combined with movement analogous to an agonistic approach
	Push [12,13,37,54,56,58,89,92,94]	Pressing of the head, neck, shoulder, body, or croup against another in an apparent attempt to displace the target animal		9	
	Strike [37,54,55,56,58,64,91,92]	A rapid motion of one or both forelegs in the anterior direction	Arched neck threat and posturing [37,58]	8	
	Strike-threat [37,64]	The aggressor’s ears are laid back, and its head and shoulders are oriented toward another individual. One or both forelimbs move out- and forward toward the other animal, but no contact is made		2	
**Agonistic submissive**	Avoidance/Withdrawal [14,22,26,30,37,64,90,92]	Movement that maintains or increases the distance to an approaching horse (which does not threaten). While making way, the subordinate usually lays its ears back	Only head turn away from the initiator [14,30]	8	These three terms are used interchangeably → clarification of the definitions is required. We propose using avoidance/withdrawal to indicate increase/maintenance of distance to a non-threatening approaching horse, retreat as a reaction to an agonistic approach at the walk or trot, and flight as a rapid increase/maintenance of distance in response to an attack
	Retreat [27,34,37,41,52,80]	One individual immediately moves away from an animal that approaches to within 2 m of it to maintain or increase the distance		6	
	Flight [22,56]	Avoiding, retreating from another horse, usually with ears laid back	Walking, trotting or galloping [56]	2	
	Balk [37]	Abrupt halt or reversal of direction with movement of the head and neck in a rapid sweeping dorsolateral motion away from an apparent threat while the hind legs remain stationary. The forelegs may simultaneously lift off the ground		1	
	Snapping [5,12,13,22,37,54,55,58,79]	Corners of the open mouth are pulled back, showing teeth and gums, making chewing motions. Hindlegs may be slightly bent in a cringing position. Head and neck are extended, the ears are oriented back or laterally	An appeasement act delivered to older/higher-ranked animals [55]	9	
**affiliative**	(Affiliative) Approach [5,14,27,30,55,64,80,90,93]	Moving to within 1 m [5]/1 [27]/2 [14,26,30] body-lengths of another horse that does not immediately move away and staying there for at least 5 [64]/10 [14,26,30] s without agonistic interaction	Across one [27]/two [14,26,30] body-length distance	9	
	Grooming approach [34,41,52]	Approach with subsequent mutual grooming		3	
	Mutual approach [34,41,52]	Both animals approach each other		3	
	Following [14,26,30,37,55,56,79,90]	Moving immediately behind another horse that had just initiated locomotion and stay within three body-lengths for at least 10 s without agonistic interaction and without initiating physical contact	Head low without any attempt to attack or bite [56]	8	
	Friendly body contact/Touching [5,14,26,30,55,79,89,92]	Touch made with ears forward or laterally positioned	Lightly with the nose or lips, also called nose-body contact [55,79,89]	8	
	Head contact [37,54,58,79,90,92,93]	A position where a horse puts its chin on the back or rear of a companion		7	
	Mutual grooming [5,12,13,14,22,26,27,30,34,37,41,52,54,55,56,58,64,79,88,89,90,91,92,93,94]	Two horses stand head to tail and chew or nuzzle each other’s coats	After introductory sniffing [93], by gently nipping, nuzzling or rubbing [27,37,54,56,64,95]	25	
	Pairing/Stand resting together [26,90]	Standing together (in antiparallel position), less than 0.5–1 m apart		2	
	Pass the mane/Under the neck [92]	A horse passes (its mane) under its companion’s chin and neck. The other horse may or may not reciprocate		1	
	Play [5,12,13,22,54,55,56,58,79,89,90,92,94]	Play includes playful nips, pounces, etc. A playful character of the interaction is indicated by the ears oriented forward or laterally, lips protruded, and teeth covered. Vocalization (squeal or scream) is not produced		13	
	Play fight [12,13,54,58,94]	High-intensity play, which is reciprocated by one or more partners, includes vigorous play movements such as rearing, boxing, nipping, circling, grasping, kneeling and chasing		5	
	Rubbing with the head/chin/body [79,92]	Rubbing up and down with the forehead/cheek/chin/itself against a companion		2	
**investigative**	Head bowing [37,92]	Repeated, exaggerated, rhythmic flexing of the neck such that the muzzle is brought toward the point of the breast. Usually occurs synchronously between two horses when they first approach each other head to head	A squeal is emitted [92]	2	These behaviours can be investigative or agonistic depending on whether or not they are followed by squeals and stomping
	Nose-nose interaction [55,79,89,92]	Two horses approach each other with arched necks and touch noses standing either opposite each other or side by side	Squeal always follows, and a stomp almost always occurs [92]	4	
	Olfactory investigation (nasal/genital/body sniff) [12,13,37,54,56,58,79,90,91,92,93,94]	Sniffing various parts of another horse’s body, including the head, neck, flank, genitals, and tail or perineal region. Another horse may or may not reciprocate	Squeal produced during the behavioural ritual “sniff and squeal” [56]	11	
**neutral**	Neutral approach [34,41,52,79]	One animal approaching another without subsequent agonistic or affiliative interactions		4	

**Table 4:** Quantitative assessments of social behaviour included in the 27 papers.

Quantitative Assessment		Formula	Reference
**Frequency of Behaviours**	**Social interactions**	Total number of affiliative and agonistic behaviours observed/per horse and or per hour	[12,13,14,34,55,56,64,80,89,92,93,94]
		Mean number of social interactions per week	[58]
		Social interactions in %: (Number of observations of a behaviour/total number of observed behaviours) × 100	[5,54,55]
	**Aggressiveness, aggressive score**	Total number of agonistic behaviours observed	[27,29,79,90]
		Count of agonistic acts received and given	[55]
		Aggression rate per group per horse/total number of aggressions per group per horse	[27]
	**Activity similarity**	(Number of observations including A and B)/(Total number of observations of A + total number of observations of B)	[22,89]
		Time percentage when two horses were first neighbours and engaged in the same activity	[5]
	**Nearest neighbour**	Nearest neighbour per activity = Time that individuals were first neighbour to each other when engaged in same activity/time that individuals were first neighbour to each other × 100	[5,89]
		Frequency of two individuals being observed as “being close” or “being far”	[79]
		Number of observations including A and B/total number of observations of A + total number of observations of B	[89,92]
		Observations of an individual at a specific distance/Total observations of that individual at any distance × 100	[22,54]
		Total number of observations in which an individual was either the first or second neighbour of a particular one (single link cluster analyses)	[5]
**Duration**	**Duration of a behaviour/interaction**	Time in seconds from start to end	[37,90]
**Dominance Relationships**	**Ranking index**	Number of agonistic encounters won by A against B/total number of agonistic encounters in which A and B were involved	[27,29,34,52,55,90,92]
		Highest rank = individual with least threats possible from individuals below it	[5,55]
		Comparison between the number of threats received by individuals and the number of threats initiated	[22,91]
		[(Number of horses that this male dominates—number of horses that this male is dominated by + group size + 1)/2]	[56]

## Data Availability

Not applicable.
